# Hybrid Layers of Donor-Acceptor Copolymers with Homogenous Silver Nanoparticle Coverage for Photonic Applications

**DOI:** 10.3390/polym13030439

**Published:** 2021-01-29

**Authors:** Věra Cimrová, Sangwon Eom, Veronika Pokorná, Youngjong Kang, Drahomír Výprachtický

**Affiliations:** 1 Institute of Macromolecular Chemistry, Czech Academy of Sciences, Heyrovsky Sq. 2, 162 06 Prague, Czech Republic; pokorna@imc.cas.cz (V.P.); vyprachticky@imc.cas.cz (D.V.); 2 Department of Chemistry, Hanyang University, Seoul 04763, Korea; eoms20@naver.com (S.E.); youngjkang@hanyang.ac.kr (Y.K.); 3 Institute of Nano Science and Technology, Hanyang University, Seoul 04763, Korea

**Keywords:** hybrid layers, silver nanoparticles, donor–acceptor copolymers, perylenetetracarboxydiimide acceptor units, absorption, spectroelectrochemistry, SEM, XPS

## Abstract

Hybrid layers of donor-acceptor (D-A) copolymers containing *N,N′*-dialkylperylene-3,4,9,10-tetracarboxydiimide electron-acceptor units covered with silver nanoparticles (Ag-NPs) were prepared by electrochemical doping of pristine layers during reduction processes. In situ optical absorption spectra of the layers were recorded during the formation of Ag-NP coverage. The hybrid layers were characterized by absorption spectroscopy, scanning electron microscopy (SEM), X-ray photoelectron spectroscopy (XPS), and energy dispersive X-ray spectroscopy (EDX). In the absorption spectra of the hybrid layers, a surface plasmon band characteristic of Ag-NPs appeared. Significant improvements in light absorption due to the plasmonic effects of Ag NPs were observed. Stable Ag-NPs with an average diameter of 41–63 nm were formed on the surface, as proven by SEM and XPS. The Ag-NP coverage and size depended on the hybrid layer preparation conditions and on the copolymer composition. The metallic character of the Ag-NPs was proven by XPS. The location in the surface layer was further confirmed by EDX analysis. To the best of our knowledge, this is the first report on such hybrid layers having the potential for a variety of photonic and electronic applications.

## 1. Introduction

Third-generation semiconducting polymers, including low-bandgap donor-acceptor (D-A) copolymers, are of interest due to their many potential applications, namely in photonics and electronics, such as in light-emitting diodes, photovoltaic devices, photodetectors, organic field-effect transistors, sensors, optical switches, and electrochromic devices, due to their specific physical, optical, and/or electronic properties [1,2,3,4,5,6,7,8,9,10,11,12,13,14]. The combination of organic semiconductors and metal nanoparticles (NPs) can further improve device performances. Particularly, the incorporation of metal NPs in organic solar cells can enhance the device absorption, charge transport, and, finally, their performance [15,16,17,18].

Recently, we synthesized and characterized a series of D–A copolymers containing *N,N′*-dialkylperylene-3,4,9,10-tetracarboxydiimide electron-acceptor units, which differ by the side chains attached to the perylene-3,4,9,10-tetracarboxydiimide (PDI) units (either dodecyls or 2-ethylhexyls) and further by the electron-donor units (9,9-dioctylfluorene, 9-(2-ethylhexyl)carbazole, or 9-(heptadecan-9-yl)carbazole) [19]. We showed the effects of the alkyl side-chain combination on the photophysical and electrochemical properties and spectroelectrochemical behavior of the copolymers. Results concerning the electrochemical behavior of their thin films indicates that these copolymers could be promising for further development of interesting hybrid layers with silver nanoparticles, using electrochemical doping. The copolymers exhibit interesting photophysical and electronic properties and very good thermal and oxidation stability, which is important for the photonic and electronic applications; therefore, such hybrid layers are of research interest.

**Figure 1:**
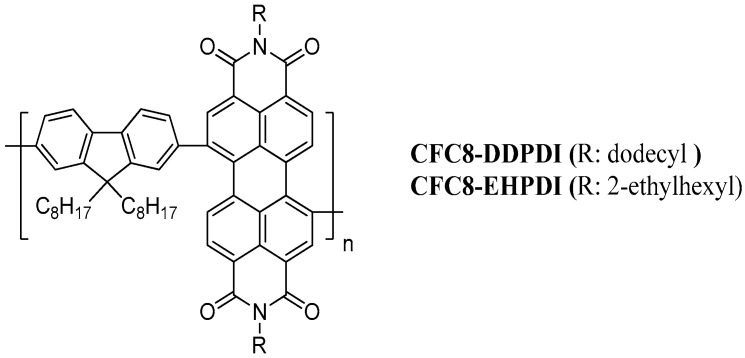
Chemical structures of the copolymers poly[*N,N′*-didodecylperylene-3,4,9,10-tetracarboxydiimide-1,7-diyl-*alt*-9,9-dioctylfluorene-2,7-diyl] (**CFC8-DDPDI**) and poly[*N,N′*-bis(2-ethylhexyl)perylene-3,4,9,10-tetracarboxydiimide-1,7-diyl-*alt*-9,9-dioctylfluorene-2,7-diyl] (**CFC8-EHPDI**).

In this work, we report the preparation and detailed characterization of hybrid layers of two PDI-based copolymers (**CFC8-DDPDI** and **CFC8-EHPDI** in Figure 1) with silver nanoparticles (Ag-NPs) of average sizes below 100 nm. The thin hybrid layers were prepared during reduction by electrochemical doping of copolymer layers, using silver nitrate. The UV–vis absorption spectra were measured in situ during the hybrid layer preparation. Scanning electron microscopy (SEM) was used to study the layer morphology and to determine the sizes of Ag-NPs. X-ray photoelectron spectroscopy (XPS) was used to analyze the surface composition of the layers to prove Ag-NP formation in the hybrid layers and to determine whether there are any interactions between the silver and copolymers. Furthermore, the layers were characterized by energy dispersive X-ray spectroscopy (EDX). To the best of our knowledge, this is the first report on such hybrid layers having great potential for a variety of photonic applications.

PDI derivatives and PDI-based polymers represent an interesting class of n-type semiconductors [20,21]. Particularly, PDI-based polymers were reported as potential n-type materials for all-polymeric solar cells [22,23,24,25]. Furthermore, they are also interesting as interfacial materials (electron transporting layers), not only in organic solar cells but also in perovskite solar cells [26,27]. Silver nanoparticles (Ag-NPs) and their composites are very interesting for photonic, as well as biological and medical, applications [28,29,30,31,32,33,34,35,36,37]. The properties of Ag-NPs are significantly different from those of bulk metals. Due to the maximization of the total surface area with Ag-NPs, the hybrid layers are particularly interesting for sensing and catalysis.

## 2. Materials and Methods

### 2.1. Materials and Layer Preparation

The copolymers under study were synthesized by the Suzuki coupling reaction and were characterized by size-exclusion chromatography, ^1^H, ^13^C NMR, and FTIR spectroscopy. The weight-average molecular weight (*M*_w_), dispersity (*Đ*), and the polymerization degree (*P*) were as follows: Mw = 27,000, *Đ* = 1.59, and *P* = 24 for **CFC8-DDPDI** and Mw = 13,200, *Đ* = 1.39, and *P* = 13 for **CFC8-EHPDI**. The synthesis of the copolymers and their characterization are described in detail in our previous paper [19]. Chloroform (spectroscopic grade), acetonitrile (extra dry), tetrabutylammonium hexafluorophosphate (electrochemical grade), and silver nitrate (AgNO_3_) were purchased from commercial suppliers (VWR International s.r.o., Stříbrná Skalice, Czech Republic, Lach-Ner, s.r.o., Neratovice, Czech Republic, Merck spol. s.r.o., Praha, Czech Republic and Sigma Aldrich spol. s.r.o., Praha, Czech Republic).

Thin layers of copolymers were prepared by spin-coating onto indium-tin oxide (ITO) substrates from chloroform solutions. The ITO glass substrates were purchased from Merck (Gernsheim, Germany). All thin-film preparations were conducted in a glove box (M. Braun Inertgas-Systeme GmbH, Garsching, Germany), under a nitrogen atmosphere Layer thicknesses were measured by using a KLA-Tencor P-10 profilometer (KLA-Tencor Corporation, Milpitas, CA, USA). The thicknesses were in the range of 130–160 nm. The hybrid layers (HLs) were prepared by electrochemical doping of pristine copolymer layers (PLs) during reduction. HL1 and HL2 were prepared during the reduction of corresponding pristine layers (PL1 and PL2) at the potentials (−1 and −1.5 V vs. Ag/Ag) exceeding those of the first and second reduction process, respectively. The electrochemical doping was performed with a PA4 polarographic analyzer (Laboratory Instruments, Prague, CZ) with a homemade cuvette three-electrode cell in a glove box, under a nitrogen atmosphere; an electrolyte solution of 0.1 M tetrabutylammonium hexafluorophosphate in anhydrous acetonitrile was used. Platinum (Pt) wire and a non-aqueous Ag/Ag^+^ electrode (Ag in 0.1 M AgNO_3_ solution) were used as the counter and reference electrodes, respectively. For the hybrid layer preparation, an electrolyte solution with AgNO_3_ (concentration 7 × 10^−^^4^ M) was used. The doping time was 12 min.

### 2.2. Methods

UV–vis spectra were measured on a Perkin-Elmer Lambda 35 UV/VIS spectrometer (PerkinElmer Instruments, Shelton, CT, USA). Spectroelectrochemical (in situ absorption spectra) measurements were performed with the homemade cuvette three-electrode cell in a glove box (M. Braun Inertgas-Systeme GmbH, Garsching, Germany) connected to a Perkin-Elmer Lambda 35 UV/VIS spectrometer, using fiber optics. A high-resolution FE-SEM (JEOL Ltd., Tokyo, Japan) JSM-7800F Prime (resolution: 0.7 nm at 15 kV) equipped with an in-lens Schottky plus field emission electron gun and an EDS detector was used for the thin-film characterization. A thin conductive layer of Pt with a thickness of ~20 Å was deposited on the films before SEM. XPS measurements were performed by using a K-Alpha^+^ XPS spectrometer (ThermoFisher Scientific, East Grinstead, UK), operating at a base pressure of 1.0 × 10^−7^ Pa. All samples were analyzed by using microfocused (spot sizes of 30 μm and 400 μm) monochromatic Al Kα X-ray radiation (72 W). Survey spectra were recorded with a step size of 1 eV and a pass energy of 200 eV, and HR spectra were recorded with a step size of 0.1 eV and a pass energy of 50 eV. The X-ray angle of incidence was 30°, and the emission angle was along the surface normal. The binding energy (BE) scale of the XPS spectrometer was calibrated by the well-known positions of the C 1s C–C and C–H, and C–O and C(=O)–O peaks of polyethylene terephthalate and the Cu 2p, Ag 3d, and Au 4f peaks of Cu, Ag, and Au metals, respectively. The data acquisition was performed by using the instrument software Thermo Avantage. The data files were converted to the AVG format. The spectra were analyzed, using CasaXPS software version 2.3.23 (Casa Software Ltd., BayHouse, UK), after data-file conversion to the VAMAS format [38]. The Universal Tougaard background (two parameters for Ag 3d and three parameters for C 1s, N 1s, and O 1s regions) was used for the analysis [39]. The analyzer transmission function, Scofield sensitivity factors, and effective attenuation lengths (EALs) for photoelectrons were applied for quantification. The analyzed results were evaluated as average values from the analysis of 3 or 4 measurements. The HR C 1s, Ag 3d, N 1s, and O 1s spectra were analyzed, using the Voigt profile. The goodness of fit was indicated by the residual standard deviation value (<1.38) and the residuals to the fit, and Monte Carlo–based error analysis was performed.

## 3. Results and Discussion

### 3.1. Hybrid Layer Preparation and Absorption

**Figure 2:**
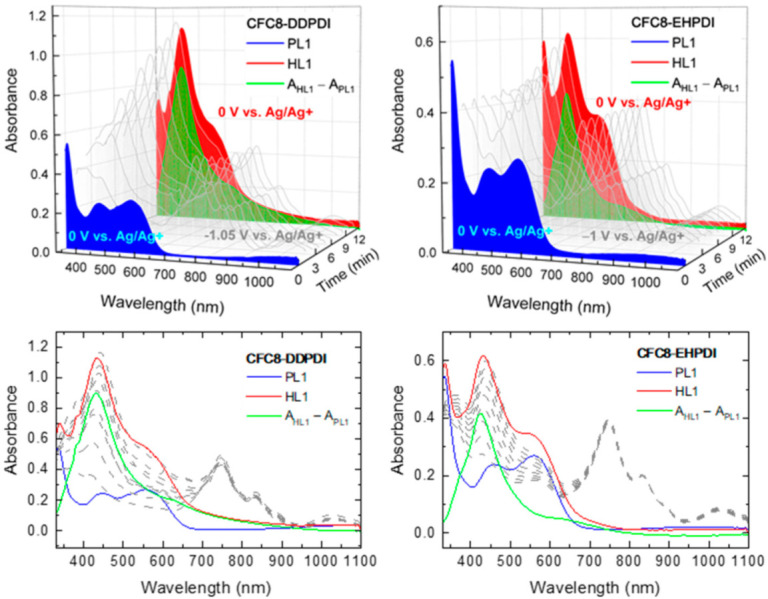
Absorption spectra of **CFC8-DDPDI** and **CFC8-EHPDI** layers on indium-tin oxide (ITO) substrates: before doping pristine (PL1—blue) and after doping hybrid (HL1—red) layers and as measured during Ag doping for 12 min at potential exceeding that of the first (−1 V vs. Ag/Ag^+^) reduction process (gray). Difference spectra (difference in the absorbance (A) of hybrid A_HL_ and pristine A_PL_ layers: A_dif_ = A_HL_ − A_PL_) are displayed by green curves.

**Figure 3:**
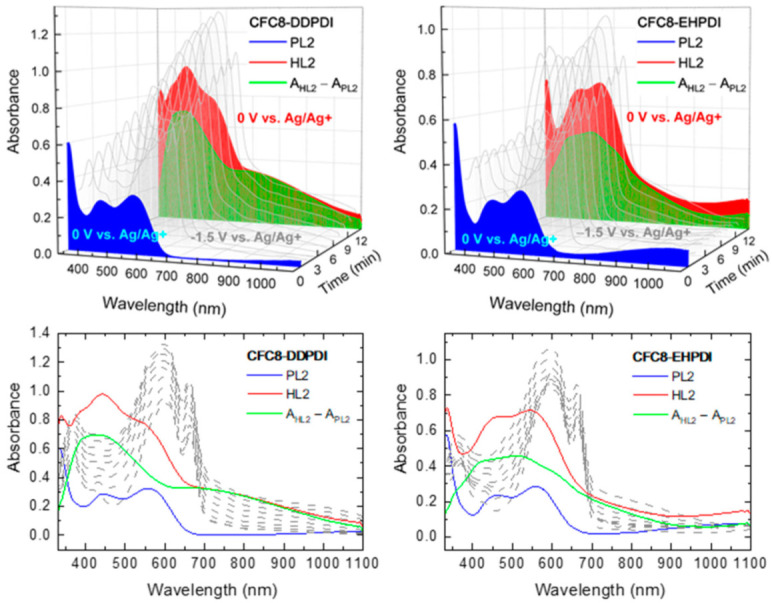
Absorption spectra of **CFC8-DDPDI** and **CFC8-EHPDI** layers on ITO substrates: before doping pristine (PL2—blue) and after doping hybrid (HL2—red) layers and as measured during Ag doping for 12 min at potential exceeding that of the second (−1.5 V vs. Ag/Ag^+^) reduction process (gray). Difference spectra (difference in the absorbance (A) of hybrid A_HL_ and pristine A_PL_ layers: Adif = A_HL_ − A_PL_) are displayed by green curves.

Thin copolymer HLs with Ag-NPs were prepared by electrochemical doping of PLs during reduction under various conditions. As reported in our previous paper, the copolymers under study (Figure 1), regardless of the copolymer structure and side chain combination, exhibited reversibility in absorption during the reduction cycle and irreversible oxidation absorption changes during the oxidation cycle. In the reduction cycle, two redox reactions are observed for all copolymers corresponding to the reduction of the carbonyl groups on the PDI unit. The first reduction, in which a carbonyl group on the PDI unit gains an electron to form PDI^−^, occurs at ca. −1 V vs. Ag/Ag^+^, and the second carbonyl group reduction to PDI^2−^ occurs at ca. −1.3 V vs. Ag/Ag^+^. Detailed electrochemical and spectroelectrochemical behavior for all copolymers is reported in our previous paper. In the reduced state, a cation dopes the negatively charged PDI unit to achieve charge neutrality. Therefore, we attempted to dope the copolymer films with silver, by adding silver nitrate (AgNO_3_), and tested whether homogenous and stable nanoparticles could be formed. The spectral changes in the absorption spectra of the **CFC8-DDPDI** and **CFC8-EHPDI** copolymer layers during Ag doping were recorded and are displayed in Figure 2 and Figure 3; these spectra were measured in situ at two potentials, −1 V and −1.5 V vs. Ag/Ag^+^, i.e., when the potential reached or exceeded the first (at −1 V vs. Ag/Ag^+^) and second (at −1.3 V vs. Ag/Ag^+^) reduction peak potentials, respectively.

The absorption spectra of the copolymer PL layers in the neutral state (blue curves in Figure 2 and Figure 3) exhibit broadband absorption in the visible region with two well-resolved maxima, which are given in Table 1. The broad bands in the visible spectral region correspond to π-π* transition of the conjugated backbone. When a potential is applied, the formation of anions and dianions with strong absorption in the visible region and NIR is observed depending on the potential value. When a potential is applied, the formation of anions and dianions with strong absorption in the visible region and NIR is observed depending on the potential value. The absorption significantly differs, as demonstrated in Figure 2 and Figure 3 (gray curves). The strong absorption band (maximum at ca. 750 nm) and additional bands with maxima at 831–836 nm and at 1024–1033 nm in the NIR are characteristic of the first reduction process corresponding to PDI^−^ anion formation (Figure 2), whereas the strong absorption in the visible region with two clear maxima at 598–603 nm and 662–665 nm corresponds to the PDI^2−^ dianion (Figure 3). Without doping, the absorption changes are reversible, i.e., when the potential is switched off, the initial absorption spectrum is recovered. Detailed electrochemical and spectroelectrochemical behavior for the copolymers is reported in our previous paper [19]. During the doping processes, an additional increase in the absorption band with a maximum at 400–450 nm is observed. This absorption band increases with increasing doping time (i.e., time of applied potential) and accompanies the absorption changes due to the reduction mentioned above. When the potential is switched off, the absorption bands corresponding to the anion and dianion disappear, and the absorption spectrum corresponding to the hybrid layer can be compared with the initial spectrum of the copolymer layer before doping. It is evident that, in both cases, the spectral changes at 400–500 nm remained and correspond to the changes produced by the formation of Ag nanoparticles, for which a surface plasmon (SP) absorption band is characteristic in this spectral region [40,41,42]. The position of the SP absorption band maximum depends on the size of the Ag-NPs.

The absorption spectrum of the pristine layer (PL) consists of broad bands with maxima at ca. 446 and 557 nm for **CFC8-DDPDI** and 460 and 559 nm for **CFC8-EHPDI**. In the spectra of the hybrid layers (HL1) prepared at the potential corresponding to PDI^−^ anion formation, the maxima of the increased absorption band corresponding to the SP band, which dominates, are located at 440 and 435 nm for **CFC8-DDPDI** and **CFC8-EHPDI**, respectively. The absorption spectra of the hybrid layers (HL2) prepared at the potential corresponding to the PDI^2−^ dianion formation show a significant absorption band contribution at long wavelengths and a long-wavelength tail. This could indicate that the Ag-NPs prepared during the first reduction process (PDI^−^ anion) not only are of smaller size (diameter) than those prepared at potentials exceeding the second reduction process potential but also differ in shape. In addition to Ag-NP spheres, Ag-NPs of other shapes, such as discs, hexagons, or triangles, could be formed [43,44]. Difference spectra (the differences in the absorbance (A) of the hybrid A_HL_ and pristine A_PL_ layers: A_dif_ = A_HL_ − A_PL_), which demonstrate improvement in light absorption due to the plasmonic effects of Ag NPs, are shown by green curves in Figure 2 and Figure 3. The absorbance enhancement contribution to the total absorbance is 0.58 and 0.74 for **CFC8-DDPDI** HL1 and HL 2 layers, respectively, and 0.40 and 0.65 for **CFC8-EHPDI** HL1 and HL2 layers, respectively. An overview of the absorption maxima for the PL and HL spectra and maxima of the difference spectra is given in Table 1. The maxima of the HL1 difference spectra at 431 and 425 nm for **CFC8-DDPDI** and **CFC8-EHPDI**, respectively, correspond well to the SP absorption of spherical Ag-NPs. The red-shifted maxima and long absorption tails in the spectra of the HL2 layers prepared during the second reduction process indicate that Ag-NPs of mixed shapes with red-shifted SP absorption are present in the HL2 layers, such as nanocircular discs with SP absorption at 510–581 nm or hexagonal and truncated nanotriangles with SP at wavelengths >581 nm [43,44]. The SP absorption peak can shift to a higher wavelength with increased aggregation of nanoparticles [45,46,47,48]. This could also be the case here due to the higher Ag-NP concentration in the HL2 layers. Broadening of the absorption peak for the HL2 layers may be attributed to the longitudinal SP peaks due to the higher amount of aggregation and formation of chain-like structures in the surface layer.

### 3.2. Scanning Electron Microscopy

**Figure 4:**
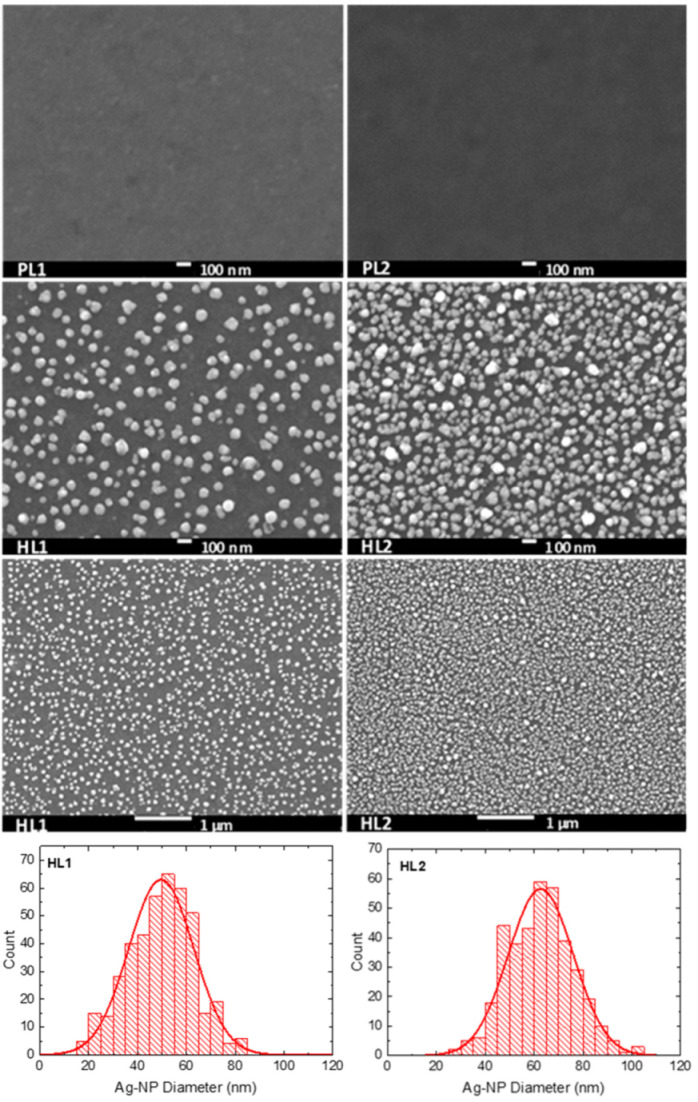
SEM images of pristine (PL1, PL2) and hybrid (HL1, HL2) thin-layer **CFC8-DDPDI** copolymer prepared at (**left**) −1.05 (HL1) and (**right**) −1.5 (HL2) V vs. Ag/Ag^+^ with histograms of the silver nanoparticle (Ag-NP) sizes and normal distribution function (Gaussian) fits.

**Figure 5:**
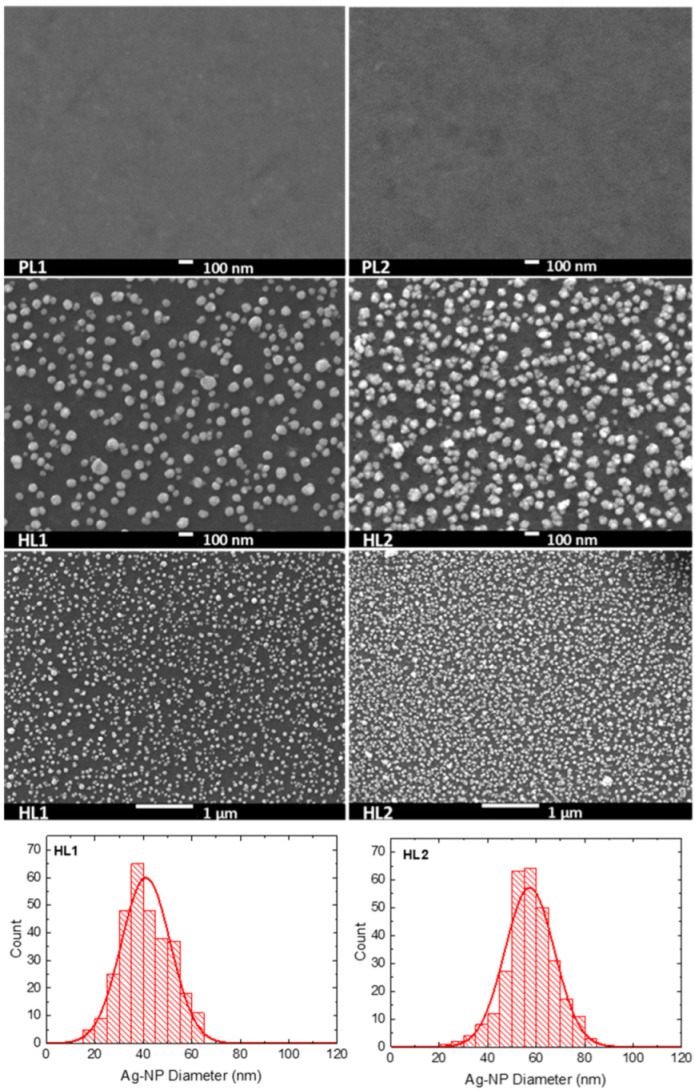
SEM images of pristine (PL1, PL2) and hybrid (HL1, HL2) thin-layer **CFC8-EHPDI** copolymer prepared at (**left**) −1 (HL1) and (**right**) −1.5 (HL2) V vs. Ag/Ag^+^ with histograms of the Ag-NP sizes and normal distribution function (Gaussian) fits.

The surface morphology was studied by SEM, to determine the size and distribution of the Ag-NPs in the hybrid layers. SEM images of pristine and hybrid copolymer thin layers made of **CFC8-DDPDI** and **CFC8-EHPDI** are shown in Figure 4 and Figure 5, demonstrating the presence of Ag-NPs and their good homogenous coverage. Ag-NP sizes were extracted from the SEM micrographs by using ImageJ software, and histograms with normal distribution function (Gaussian) fits are also displayed in the figures. The general formula for the probability function of the normal distribution is given by the equation *f*(*x*) = *f*_0_ + *A* exp(−(*x* − *d*_0_)^2^/2*σ*^2^). The mean particle diameter, *d*_0_, and distribution parameter, σ, of the distribution function are given in Table 1, together with the absorption maxima of the hybrid layers. Smaller mean particle diameters (average size) of Ag-NPs were evaluated for the HL1 hybrid layers prepared during the first reduction process, whose distribution was also narrower than that for the HL2 layer prepared at a potential exceeding the second reduction potential. The smaller diameters correspond well to the absorption maxima at shorter wavelengths, which agrees with SP theory [40]. Other shapes than spheres and chain-like structures were also identified on the HL2 layer surfaces, which is in good agreement with the absorption spectra outlined above.

### 3.3. X-ray Photoelectron Spectroscopy

**Figure 6:**
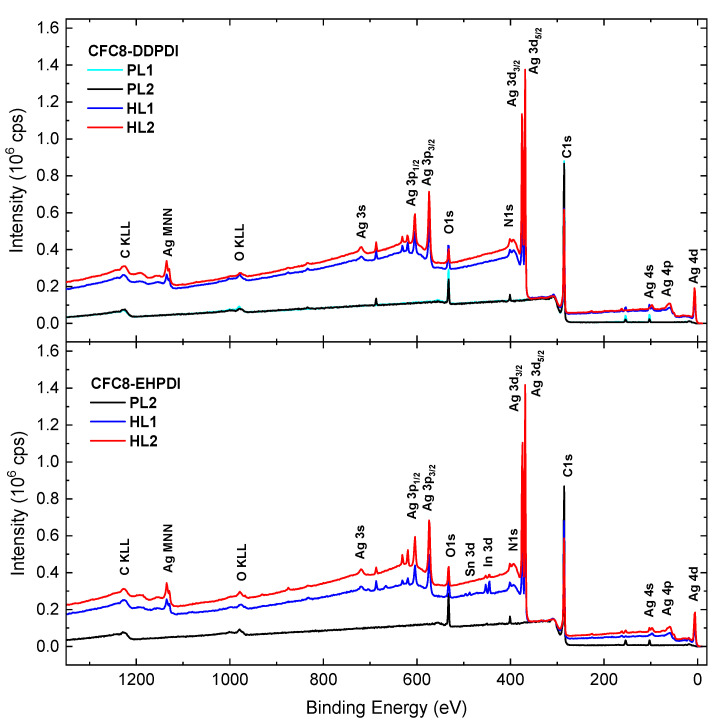
XPS wide (survey) spectra of the pristine and hybrid layers of **CFC8-DDPDI** and **CFC8-EHPDI** copolymers prepared at −1 (HL1) and −1.5 (HL2) V vs. Ag/Ag^+^.

XPS as a surface analytical method was used to prove the Ag-NP presence at the surface of the hybrid layers and to determine the changes in the structure of the pristine (PL1, PL2) and hybrid (HL1, HL2) layers. The measurements were performed on the same layer, its pristine part, and hybrid part containing Ag-NPs. The XPS wide (survey) spectra of the PL and HL layers for both copolymers **CFC8-DDPDI** and **CFC8-EHPDI** shown in Figure 6 contain signals from all constituent elements (C, O, N) of the copolymers. In addition, Ag characteristic peaks corresponding to Ag 4d, Ag 4p, Ag 4s, Ag 3d_5/2_, Ag 3d_3/2_, Ag 3p_3/2_, Ag 3p_1/2_, and Ag 3s photoelectron and Ag MNN Auger lines [49] are observed in the spectra of the hybrid layers, proving successful coverage of the copolymer surface by Ag-NPs, as shown by SEM. Minor In and Sn 3d peaks appear in the spectra of **CFC8-EHPDI** HL layers. They originate from the ITO substrate and are caused by free pin holes in the layer. The increase in the intensity of Ag peaks is accompanied by a decrease in the C 1s, N 1s, and O 1s peak intensities. Visual inspection of the XPS survey spectra is important, particularly for the hybrid layers, where the inelastic background increased with increasing Ag-NP coverage due to inelastically scattered photoelectrons [50].

Quantitative XPS analysis is complicated by the elastic and inelastic scattering processes that occur during the transport of the excited electrons out of the solid. In the first approach, the traditional quantification analysis, in which the surface concentration is proportional to the measured peak intensity (or peak area considering the proper relative sensitivity factors (RSFs)), was used to obtain information about surface atomic element concentrations. The quantification was performed by using Casa XPS software considering the sum of C, N, O, and Ag atoms for the PL and HL layers as 100%. Regions up to approximately 30 eV below the peak kinetic energy (KE) were used for the evaluation. The results are summarized in Table 2. The quantification analysis revealed slightly higher amounts of oxygen than the values expected for the stochiometric ratio of the repeat copolymer unit (expected C:N:O is 77:2:4 for **CFC8-DDPDI** and 69:2:4 for **CFC8-EHPDI**). The higher O values (up to seven or eight atoms instead of four) arise predominantly from ambient atmosphere (air/water) contamination and, for the HL1 **CFC8-EHPDI** layer, partly from the ITO substrate, as mentioned above. Contaminating species (water and ITO) were determined from the detailed analysis of O 1s high-resolution spectra measured on several thin films and at different spots, as shown below. The evaluated atomic Ag percentage values up to 20% for the HL2 hybrid layers prepared at the potential of −1.5 V vs. Ag/Ag^+^, which exceeds the second reduction process potential corresponding to the PDI^2^^−^ dianion formation, are higher than the Ag atomic percentage values of 12–15% for the HL1 layers prepared at the potential of −1 up to −1.05 V vs. Ag/Ag^+^ corresponding to the PDI^−^ anion formation. The higher Ag contents for HL2 than for HL1 layers correlate well with the SEM results.

The C 1s, N 1s, and O 1s peak area intensity ratios of the HL and PL layers ($I_{C1s}^{HL}/I_{C1s}^{PL}$, $I_{N1s}^{HL}/I_{N1s}^{PL}$, $I_{O1s}^{HL}/I_{O1s}^{PL}$) characterizing the peak intensity attenuation and the ratios of the RSF-corrected peak intensities of Ag 3d and C 1s ($I_{Ag3d}^{HL}/I_{C1s}^{HL}$) for the HL layers showing an Ag increase in HL are summarized in Table 3. We also performed a comparison of the HL spectra with a reference metallic Ag spectrum. The metallic Ag reference spectrum was taken on metallic Ag cleaned by argon ion sputtering until no O or C contaminants could be observed. The intensity ratios of HL and metallic Ag, $I_{Ag3d}^{HL}/I_{Ag3d}^{Metal}$, displayed in Table 3, are qualitatively in good agreement with the Ag atomic percentage determined from traditional analysis. In addition to the traditional analysis, we also evaluated the total amount of substance (AOS) within the outermost few nanometers of a surface, by analyzing the XPS spectrum, using the simple algorithm suggested by Tougaard [51,52]. The Ag atomic percentages obtained by using this method (Table 3) are slightly higher than those evaluated by using traditional quantification analysis.

**Figure 7:**
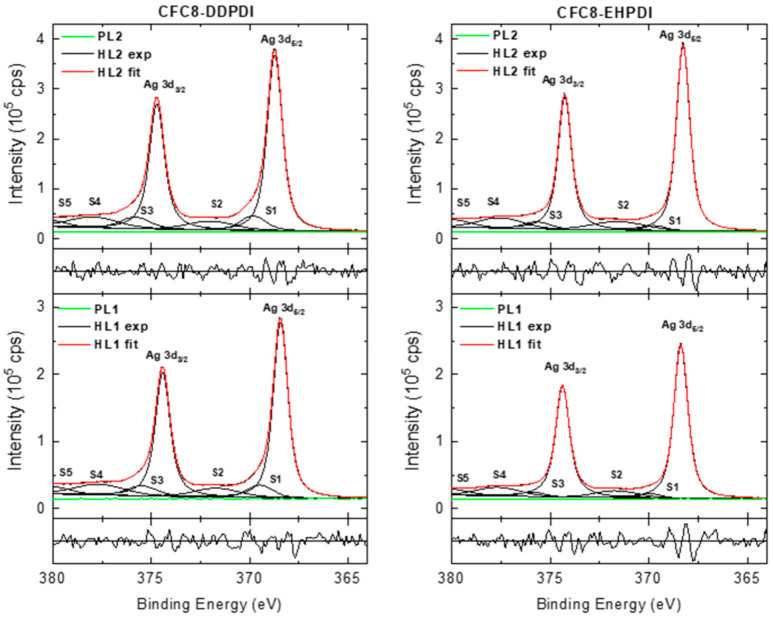
High-resolution Ag 3d spectra of **CFC8-DDPDI** (**left**) and **CFC8-EHPDI** (**right**) layer surfaces, including components and normalized residuals displayed below the spectra.

**Figure 8:**
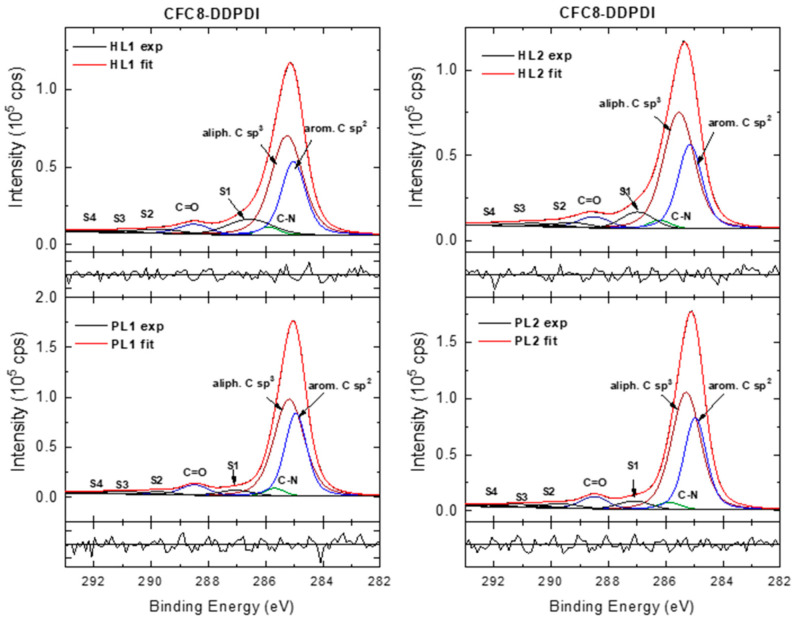
High-resolution C 1s spectra of **CFC8-DDPDI** pristine (PL1, PL2) and hybrid (HL1, HL2) layer surfaces, including components and normalized residuals displayed below the spectra.

**Figure 9:**
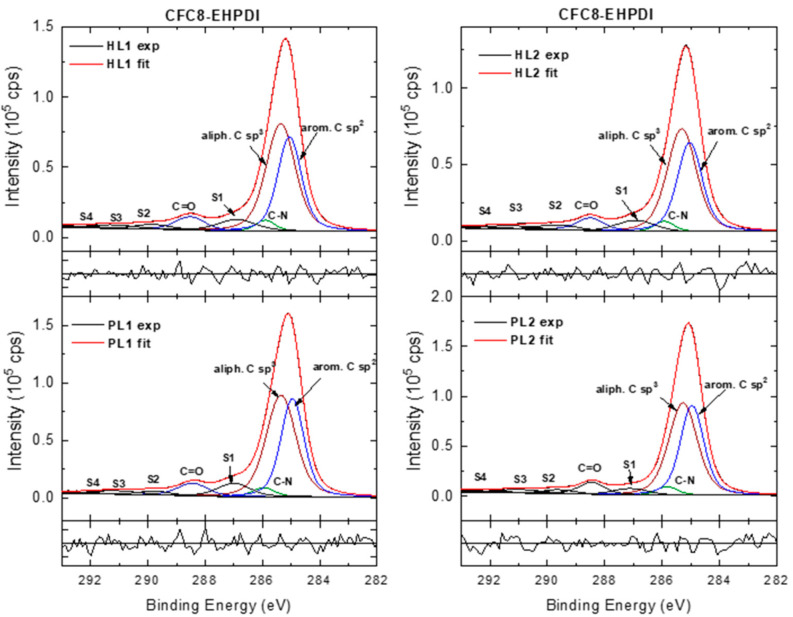
High-resolution C 1s spectra of **CFC8-EHPDI** pristine (PL1, PL2) and hybrid (HL1, HL2) layer surfaces, including components and normalized residuals displayed below the spectra.

**Figure 10:**
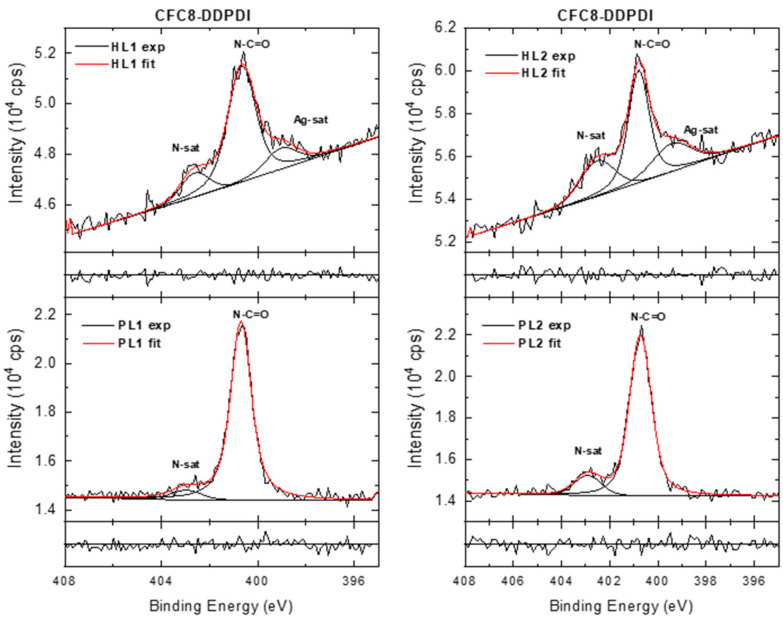
High-resolution N 1s spectra of **CFC8-DDPDI** pristine (PL1, PL2) and hybrid (HL1, HL2) layer surfaces, including components and normalized residuals displayed below the spectra.

**Figure 11:**
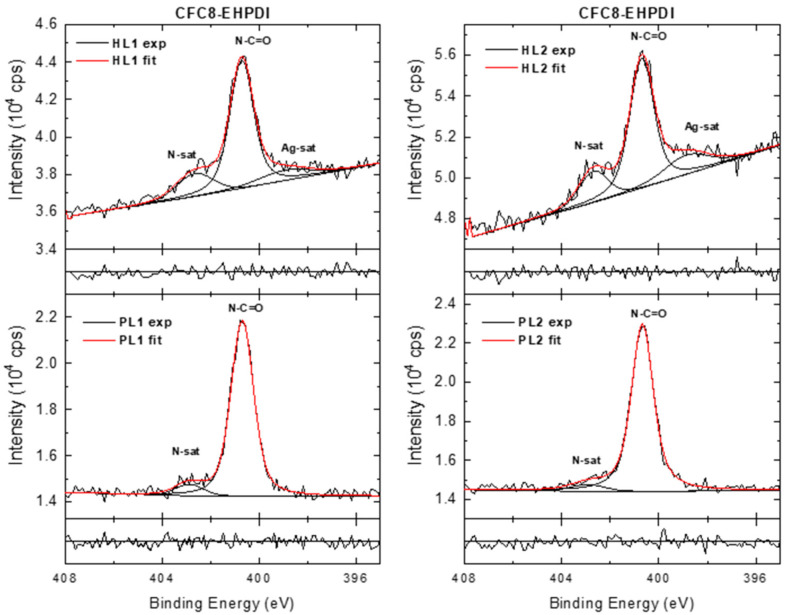
High-resolution N 1s spectra of **CFC8-EHPDI** pristine (PL1, PL2) and hybrid (HL1, HL2) layer surfaces, including components and normalized residuals displayed below the spectra.

**Figure 12:**
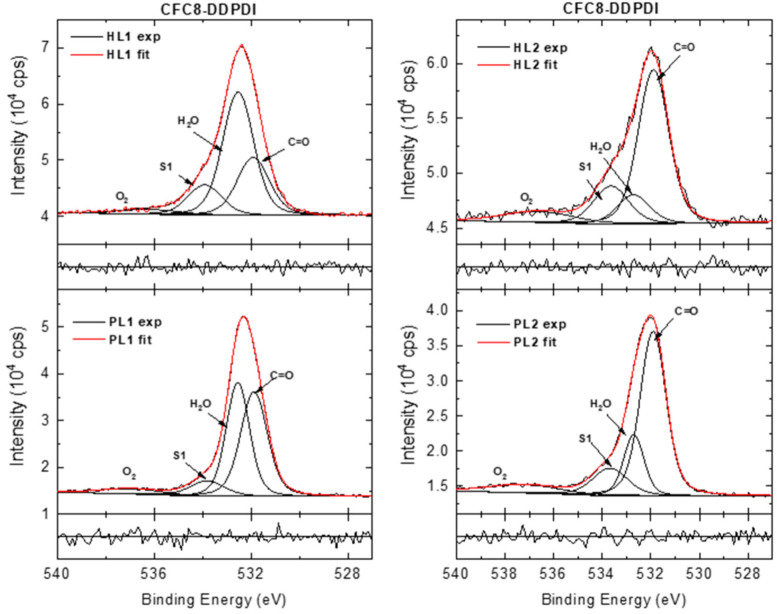
High-resolution O 1s spectra of **CFC8-DDPDI** pristine (PL1, PL2) and hybrid (HL1, HL2) layer surfaces, including components and normalized residuals displayed below the spectra.

**Figure 13:**
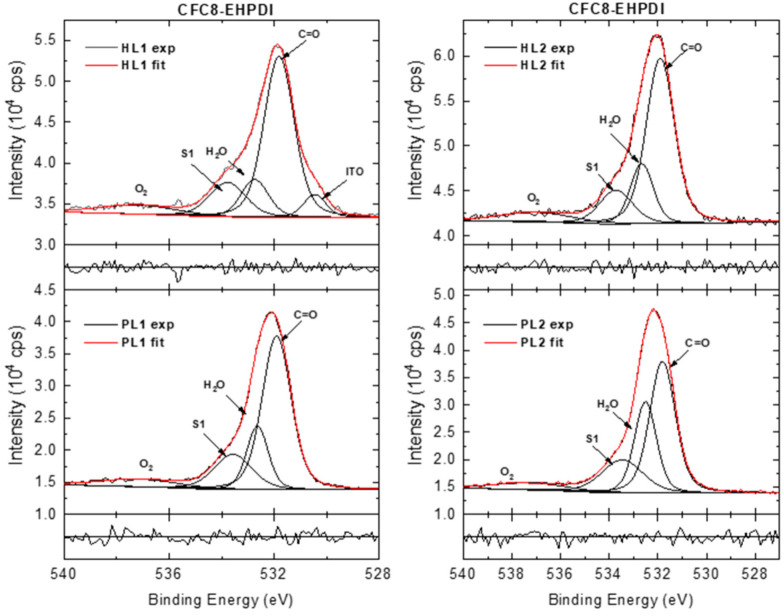
High-resolution O 1s spectra of **CFC8-EHPDI** pristine (PL1, PL2) and hybrid (HL1, HL2) layer surfaces, including components and normalized residuals displayed below the spectra.

The high-resolution (HR) Ag 3d, C 1s, N 1s, and O 1s spectra showed more details. Representative HR spectra of pristine and hybrid layer surfaces are shown in Figure 7, Figure 8, Figure 9, Figure 10, Figure 11, Figure 12 and Figure 13. The HR Ag 3d spectra of the hybrid layers exhibit characteristic Ag 3d_5/2_–Ag 3d_3/2_ spin–orbit components with a spin–orbit splitting separation of 6 eV, whereas no peaks originating from silver appear in the Ag 3d spectra of the pristine copolymer layers. The spin–orbit splitting separation of 6 eV is in good agreement with the value for metallic Ag [53]. The BEs of Ag 3d_5/2_ in all Ag 3d spectra of the hybrid layers are in good agreement or slightly up-shifted compared with the Ag 3d_5/2_ BE of metallic Ag (368.28 eV). The up-shifts were detected in the spectra of the **CFC8-DDPDI** layers, with the most pronounced up-shift of 0.44 eV for the HL2 layers. Both the Ag 3d_5/2_ and Ag 3d_3/2_ main peaks show asymmetry; therefore, the HR Ag 3d spectra (Figure 7) have to be modeled with additional symmetrical lines corresponding to the satellites, similar to the metallic Ag spectrum. The satellites (S1, S3) with a 1–1.5 eV energy separation relative to the main Ag 3d_5/2_ and Ag 3d_3/2_ peaks can be interpreted as an emission process associated with an atomic-like pure p-screening, two other satellites (S2 and S4 ca. 3.5 eV) are associated with the plasmon loss features, and the fifth satellite (S5 ca. 12 eV) is due to the 4d → 5p shake-up [54,55,56].

NP-size-dependent or Ag-coverage-dependent up-shifts in the Ag 3d BE have been observed before for Ag-NPs or clusters, but for significantly smaller sizes than in our case [57,58,59,60,61,62,63]. Ag BE up-shifts have also been observed on a rubene layer covered with thin Ag, explained by the negative charge transfer from diffused Ag to the rubene molecules [64], and further in Ag-NP–conducting polymer polyaniline, explained by partial oxidation of Ag due to the interaction with N atoms [65]. In our case, the Ag-NPs are in the Ag metallic state, which was proven by the values of the Auger parameter (AP). The Ag metallic state is distinguishable mainly through the AP, which is nearly 2 eV higher than the AP value for any other oxidized Ag species [66]. The modified AP α is defined as the sum of the BE and the kinetic energy (KE) of prominent and convenient photoelectron and Auger peaks, respectively, from the same element in an XPS spectrum. The APs (α_4_, α_5_) were evaluated from the experimental BE of the Ag 3d_5/2_ component maximum (BE_Ag3d_) and the KE of the two most intense peaks existing in the Auger electron structure M_4_N_45_N_45_ (α_4_) and M_5_N_45_N_45_ (α_5_) for HL copolymer layers, metallic Ag and AgNO_3_ samples. The AP values for the hybrid layers (α_4_ = 725.9–726.0 and α_5_ = 720.3–720.6 eV) are in very good agreement with the evaluated values for metallic Ag and are significantly different from the values for Ag in other chemical states. Here, we demonstrated this fact by comparison with AgNO_3_ (α_4_ and α_5_ values of 723.0 and 718.2 eV, respectively). Thus, a chemical reaction between Ag and the O or N atoms of the copolymer can be ruled out. A possible explanation for the BE up-shift could be charge redistribution at the surface and interface [67].

Modeling the C 1s core-level spectra is more complicated than that of the Ag spectra because there is a set of chemically shifted components due to the different chemical states of carbons with different BEs in the copolymer structure. There are more bonding environments for carbon, for which the shift is small and difficult to resolve. In this work, the HR C 1s spectra were modeled with four components corresponding to aromatic C sp^2^, aliphatic C sp^3^, C–N sp^3^, and C=O groups and π-π* satellite components (Figure 8 and Figure 9).

The component areas were constrained to correspond to the chemical structure of the polymer repeat units. In the HR spectra of the pristine PL layer, the component peak maxima located at 284.9 eV (aromatic C sp^2^), 285.3 eV (aliphatic C sp^3^), 286 eV (C–N sp^3^), and 288.5 eV (O=C–N) are in good agreement with the positions reported in the literature for the corresponding groups [68,69,70]. The peak at approximately 287 eV can be assigned to the shake-up excitations associated with the aromatic C sp^2^. Its position agrees with that observed in the spectra of PDI derivatives [71,72]. The other weaker peaks at higher BEs are correlated with shake-up phenomena-π-π* satellite peaks associated with C=O and aromatic C sp^2^ [70,71,72,73]. In the spectra of the hybrid layers, the main peak area decreases, and an increase in the satellite/main peak area ratios is observed. The most pronounced changes accompanied by up-shift of the main peaks were found in the **CFC8-DDPDI** HL2 spectrum.

In the HR N 1s spectra of the pristine layers (Figure 10 and Figure 11), the dominant peak at 400.7 eV is assigned to N–C=O groups, and the smaller peak at higher BE (at approximately 403 eV) represents π-π* satellite features characteristic of nitrogen-containing aromatic polymers. In the HR N 1s spectra of the hybrid layers, the peak corresponding to N–C=O groups dominates, but the analysis of the spectra is more complicated. It is evident from the wide spectra that the N 1s energy region is influenced by the Ag 3d photoelectron losses. An additional peak appears at a lower BE, and its contribution increases with increasing Ag 3d doublet peaks. This peak could be assigned to the Ag 3d core line satellite (bulk plasmon) [54]. All changes can be directly correlated with the presence of metallic Ag-NPs on the surface of the copolymer film. An NO^3^^−^ contribution (406.1 eV) was not observed in the HR N 1s spectra.

The HR O 1s spectra of the **CFC8-DDPDI** layers were deconvoluted into four components (Figure 12). The main contribution with a maximum at 531.9 eV is assigned to the C=O of the imide groups in the copolymer and the peak at 533.9 eV to the shake-up [74]. The components with maxima at approximately 532.7 and 537 eV could originate from air contamination, from adsorbed water and oxygen, respectively [75,76,77,78]. The air contamination content could be different for different layers, as demonstrated by comparison of the PL1 and PL2 layers. The higher relative area of the peak corresponding to the adsorbed water in the PL1 spectrum than that in the PL2 spectrum is in good agreement with the quantitative analysis, where a higher amount of oxygen was evaluated for PL1 than for PL2. An additional peak at 530.3 eV appears in the HR O 1s spectra of the **CFC8-EHPDI** films (Figure 13), which can be assigned to the indium tin oxide from the ITO substrate [79]. The In and Sn appearances are evident in the survey spectra (In 3d and Sn 3d doublet peaks). In the HR O 1s spectra of the hybrid layers, an increase in the satellite/main peak area ratio is observed in addition to a decrease in the main peak area and a decrease in the adsorbed H_2_O peak area.

### 3.4. Energy Dispersive X-ray Spectroscopy

**Figure 14:**
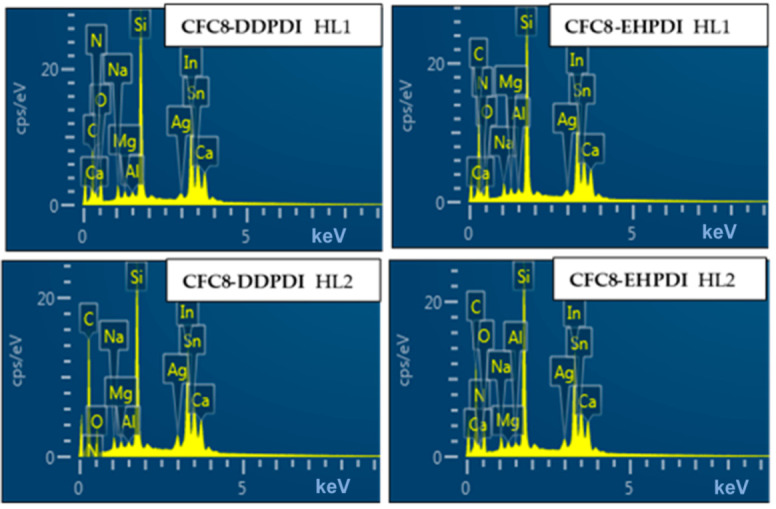
EDX pattern of the **CFC8-DDPDI** (**left**) and **CFC8-EHPDI** (**right**) hybrid layers.

**Figure 15:**
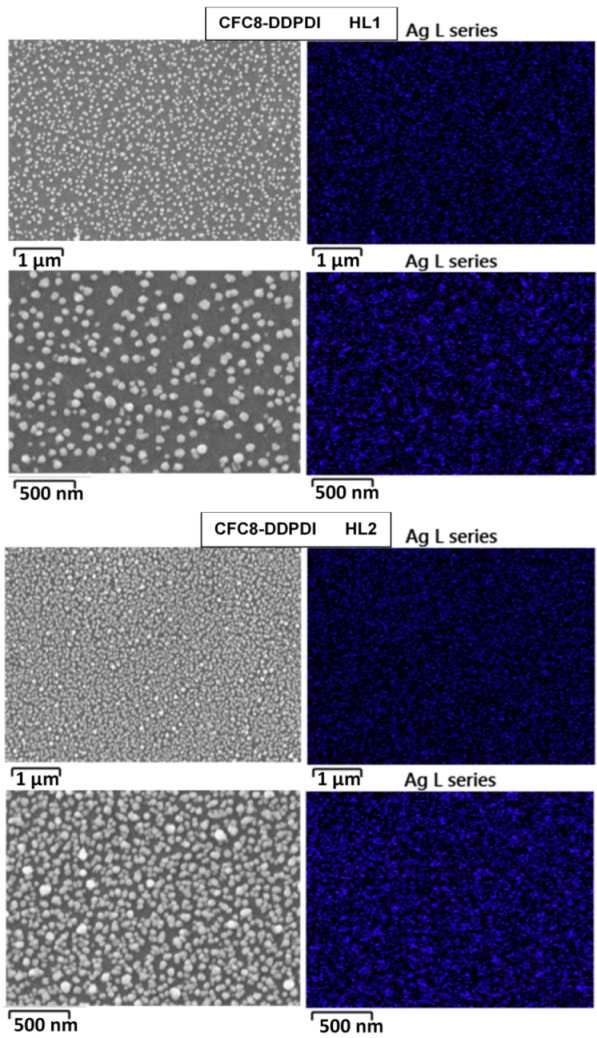
Electron (**left**) and elemental (**right**) mapping images of **CFC8-DDPDI** HL1 and HL2 hybrid layers.

**Figure 16:**
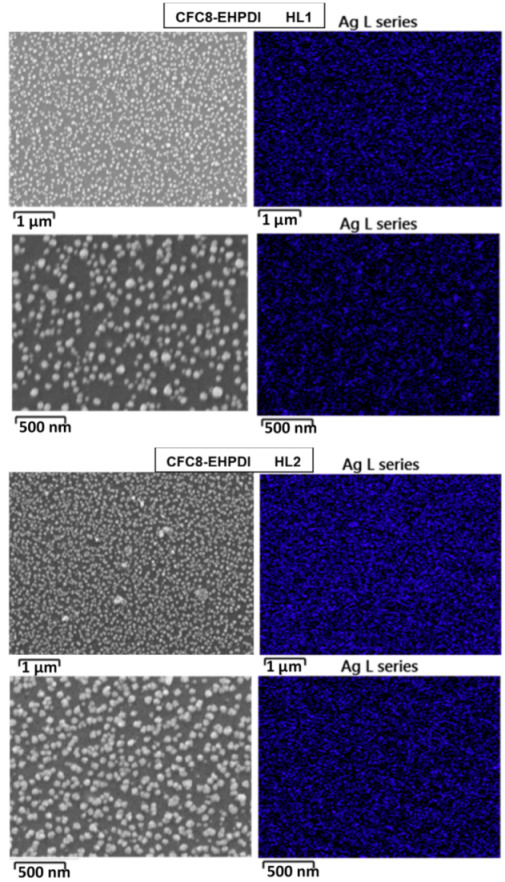
Electron (**left**) and elemental (**right**) mapping images of **CFC8-EHPDI** HL1 and HL2 hybrid layers.

EDX analysis, which allows inspection of the spatial distribution of elements, also confirms the Ag-NP presence in the hybrid layers. EDX pattern and an example of elemental mapping images are shown in Figure 14, Figure 15 and Figure 16. Peaks corresponding to the C, O, N, and Ag atoms appear in the EDX spectrum of the hybrid layers. Additional strong peaks, such as the In, Sn, O, Na, Mg, Al, Si, and Ca peaks originating from the ITO glass substrate, are present because the EDX technique analyzes the X-ray characteristic radiation emitted upon bombarding the sample with electrons, which originates from depths up to 10 µm underneath the surface. The Ag percentage values determined from EDX analysis (atomic % considering as 100% the sum of all elements) and the Ag/C intensity peak ratio values given in Table 4 are significantly lower than the values determined from XPS analysis, which proves that the Ag-NPs are located at the surface layer. The relative values of the EDX results (normalized $I_{Ag3d}^{HL}/I_{C1s}^{HL}$) for the HL1 and HL2 layers are in very good agreement with the relative values obtained from XPS analysis (normalized $I_{Ag}^{HL}/I_{C}^{HL}$) and correlate well with the SEM results.

## 4. Conclusions

Hybrid layers of the PDI-based **CFC8-DDPDI** and **CFC8-EHPDI** copolymers with Ag-NPs were prepared during reduction by electrochemical doping of pristine layers, using silver nitrate. The hybrid layers were characterized by optical, SEM, XPS, and EDX methods. The formation of Ag-NPs was evident in the measured in situ optical absorption spectra, where a characteristic SP absorption band appeared. Hybrid layers exhibited significantly higher light absorption due to the plasmonic effects of Ag-NPs, which is promising for the use in solar cells. The presence of Ag-NPs and their size were proven by SEM, XPS and EDX methods. The results of the SEM study showed very homogenous coverage of Ag-NPs, with the average diameter depending on the potential applied during reduction and on the copolymer side chains. Larger diameters (63 and 57 nm for **CFC8-DDPDI** and **CFC8-EHPDI,** respectively) were determined for the HL2 layers prepared at potentials corresponding to the second reduction than those (50 and 41 nm) for the HL1 layers prepared at potentials corresponding to the first reduction process. XPS confirmed the existence of stable Ag-NPs and their metallic character. The Ag atomic percentage was higher for HL2 than for HL1 layers. EDX analyses confirmed the location of the Ag-NPs in the surface layer. The hybrid layers with Ag-NPs of diameters below 100 nm have the potential for various photonic and electronic applications, particularly in photovoltaics, sensing, or catalysis.

## Figures and Tables

**Table 1:** Absorption maxima of the pristine and hybrid layers (*λ*_max_), maxima of difference spectra (*λ*_difmax_), and parameters of the distribution (*d*_0_, *σ*) determined from SEM. The main maxima in the visible spectral region are printed in bold.

Copolymer	Layer	*λ*_max_ (nm)	*λ*_difmax_ (nm)	*d*_0_ (nm)	*σ* (nm)
**CFC8-DDPDI**	PL1	336, 447, **556**			
**CFC8-DDPDI**	PL2	334, 445, **557**			
**CFC8-DDPDI**	HL1	340, **433**	431	49.7	13.4
**CFC8-DDPDI**	HL2	338, **441**	439	62.7	13.4
**CFC8-EHPDI**	PL1	334, 460, **559**			
**CFC8-EHPDI**	PL2	334, 460, **559**			
**CFC8-EHPDI**	HL1	335, **431**	425	41.0	10.1
**CFC8-EHPDI**	HL2	336, 472, **546**	514	57.3	10.2

**Table 2:** Results of XPS traditional quantitative analysis (atomic %) for the copolymer layers under study.

Copolymer	Layer	Ag 3d	C 1s	N 1s	O 1s
**CFC8-DDPDI**	PL1	0	89.5 ± 0.4	2.3 ± 0.3	8.2 ± 0.3
**CFC8-DDPDI**	HL1	15.0 ± 0.2	75.2 ± 0.7	1.9 ± 0.6	7.9 ± 0.6
**CFC8-DDPDI**	PL2	0	91.0 ± 0.4	2.4 ± 0.3	6.6 ± 0.3
**CFC8-DDPDI**	HL2	20.3 ± 0.3	72.9 ± 0.7	1.9 ± 0.6	4.9 ± 0.5
**CFC8-EHPDI**	PL1	0	90.1 ± 0.4	2.6 ± 0.3	7.3 ± 0.3
**CFC8-EHPDI**	HL1	11.7 ± 0.2	79.4 ± 0.6	2.3 ± 0.5	6.6 ± 0.5
**CFC8-EHPDI**	PL2	0	89.6 ± 0.4	2.6 ± 0.3	7.8 ± 0.3
**CFC8-EHPDI**	HL2	19.0 ± 0.3	72.0 ± 0.7	2.1 ± 0.6	6.9 ± 0.6

**Table 3:** XPS results as ratios of relative sensitivity factor (RSF)-corrected peak intensities and Ag atomic percentage evaluated by using the simple algorithm suggested by Tougaard [51,52], for the copolymer layers under study.

Copolymer	Layer	$I_{C1s}^{HL}/I_{C1s}^{PL}$	$I_{N1s}^{HL}/I_{N1s}^{PL}$	$I_{O1s}^{HL}/I_{O1s}^{PL}$	$I_{Ag3d}^{HL}/I_{C1s}^{HL}$	$I_{Ag3d}^{HL}/I_{Ag3d}^{Met}$	Ag at. %
**CFC8-DDPDI**	HL1	0.702	0.702	0.811	0.200	0.134	16.3
**CFC8-DDPDI**	HL2	0.694	0.693	0.647	0.279	0.186	21.7
**CFC8-EHPDI**	HL1	0.819	0.819	0.849	0.148	0.116	13.5
**CFC8-EHPDI**	HL2	0.656	0.656	0.718	0.265	0.173	20.2

**Table 4:** EDX analysis results and comparison with XPS results for the hybrid layers under study.

Copolymer	Layer	EDX	XPS	EDX	XPS	EDX
		Ag Atomic %	$I_{Ag3d}^{HL}/I_{C1s}^{HL}$	$I_{Ag}^{HL}/I_{C}^{HL}\;$	${\mathrm{Normalized}}_{\;}I_{Ag3d}^{HL}/I_{C1s}^{HL}$	${\mathrm{Normalized}}_{\;}I_{Ag}^{HL}/I_{C}^{HL}\;$
**CFC8-DDPDI**	HL1	0.51	0.200	0.0120	0.715	0.742
**CFC8-DDPDI**	HL2	1.00	0.279	0.0162	1	1
**CFC8-EHPDI**	HL1	0.45	0.148	0.0082	0.529	0.505
**CFC8-EHPDI**	HL2	0.83	0.265	0.0154	0.948	0.952

## Data Availability

Not applicable.

## References

[1] An Q.S., Zhang F.J., Gao W., Sun Q.Q., Zhang M., Yang C.L., Zhang J. (2018). High-efficiency and air stable fullerene-free ternary organic solar cells. Nano Energy.

[2] Hou J., Inganas O., Friend R.H., Gao F. (2018). Organic solar cells based on non-fullerene acceptors. Nat. Mater..

[3] Guo X., Baumgarten M., Müllen K. (2013). Designing π-conjugated polymers for organic electronics. Prog. Polym. Sci..

[4] Lv X., Li W., Ouyang M., Zhang Y., Wright D.S., Zhang C. (2017). Polymeric electrochromic materials with donor–acceptor structures. J. Mater. Chem. C.

[5] Osaka I. (2015). Semiconducting polymers based on electron-deficient π-building units. Polym. J..

[6] Wu J.S., Cheng S.W., Cheng Y.J., Hsu C.S. (2015). Donor-acceptor conjugated polymers based on multifused ladder-type arenes for organic solar cells. Chem. Soc. Rev..

[7] Xue R., Zhang J., Li Y., Li Y. (2018). Organic Solar Cell Materials toward Commercialization. Small.

[8] Yuan J., Ouyang J.Y., Cimrova V., Leclerc M., Najari A., Zou Y.P. (2017). Development of quinoxaline based polymers for photovoltaic applications. J. Mater. Chem. C.

[9] Cao K., Shen D.E., Österholm A.M., Kerszulis J.A., Reynolds J.R. (2016). Tuning Color, Contrast, and Redox Stability in High Gap Cathodically Coloring Electrochromic Polymers. Macromolecules.

[10] Lee C., Lee S., Kim G.-U., Lee W., Kim B.J. (2019). Recent Advances, Design Guidelines, and Prospects of All-Polymer Solar Cells. Chem. Rev..

[11] Root S.E., Savagatrup S., Printz A.D., Rodriquez D., Lipomi D.J. (2017). Mechanical Properties of Organic Semiconductors for Stretchable, Highly Flexible, and Mechanically Robust Electronics. Chem. Rev..

[12] Zhan X., Tan Z., Domercq B., An Z., Zhang X., Barlow S., Li Y., Zhu D., Kippelen B., Marder S.R. (2007). A high-mobility electron-transport polymer with broad absorption and its use in field-effect transistors and all-polymer solar cells. J. Am. Chem. Soc..

[13] Jensen J., Hösel M., Dyer A.L., Krebs F.C. (2015). Development and Manufacture of Polymer-Based Electrochromic Devices. Adv. Funct. Mater..

[14] Cimrová V., Výprachtický D., Pokorná V. (2019). Donor-acceptor copolymers containing bithiophene and dithiophenylthienothiadiazole units with fast electrochromic response. J. Mater. Chem. C.

[15] Maake P.J., Bolokang A.S., Arendse C.J., Vohra V., Iwuoha E.I., Motaung D.E. (2020). Metal oxides and noble metals application in organic solar cells. Sol. Energy.

[16] Shamjid P., Abhijith T., Vivek P., Joel C.S., Reddy V.S. (2019). Plasmonic effects of Ag nanoparticles for absorption enhancement in polymer solar cells with MoO3 passivation layer. Phys. B Condens. Matter.

[17] Ginting R.T., Kaur S., Lim D.K., Kim J.M., Lee J.H., Lee S.H., Kang J.W. (2017). Plasmonic Effect of Gold Nanostars in Highly Efficient Organic and Perovskite Solar Cells. ACS Appl. Mater. Interfaces.

[18] Cheng Z.K., Javed N., O’Carroll D.M. (2020). Optical and Electrical Properties of Organic Semiconductor Thin Films on Aperiodic Plasmonic Metasurfaces. ACS Appl. Mater. Interfaces.

[19] Cimrová V., Výprachtický D., Pokorná V., Babičová P. (2019). Donor–acceptor copolymers with 1,7-regioisomers of *N,N′*-dialkylperylene-3,4,9,10-tetracarboxydiimide as materials for photonics. J. Mater. Chem. C.

[20] Jones B.A., Facchetti A., Wasielewski M.R., Marks T.J. (2007). Tuning orbital energetics in arylene diimide semiconductors. Materials design for ambient stability of n-type charge transport. J. Am. Chem. Soc..

[21] Russ B., Robb M.J., Brunetti F.G., Miller P.L., Perry E.E., Patel S.N., Ho V., Chang W.B., Urban J.J., Chabinyc M.L. (2014). Power Factor Enhancement in Solution-Processed Organic n-Type Thermoelectrics Through Molecular Design. Adv. Mater..

[22] Jiang X., Xu Y., Wang X., Yang F., Zhang A., Li C., Ma W., Li W. (2017). Conjugated polymer acceptors based on fused perylene bisimides with a twisted backbone for non-fullerene solar cells. Polym. Chem..

[23] Liu M., Yang J., Lang C., Zhang Y., Zhou E., Liu Z., Guo F., Zhao L. (2017). Fused Perylene Diimide-Based Polymeric Acceptors for Efficient All-Polymer Solar Cells. Macromolecules.

[24] Sharma S., Kolhe N.B., Gupta V., Bharti V., Sharma A., Datt R., Chand S., Asha S.K. (2016). Improved All-Polymer Solar Cell Performance of n-Type Naphthalene Diimide–Bithiophene P(NDI2OD-T2) Copolymer by Incorporation of Perylene Diimide as Coacceptor. Macromolecules.

[25] Zhou E., Cong J., Wei Q., Tajima K., Yang C., Hashimoto K. (2011). All-polymer solar cells from perylene diimide based copolymers: Material design and phase separation control. Angew. Chem. Int. Ed. Engl..

[26] Yin Z., Wei J., Zheng Q. (2016). Interfacial Materials for Organic Solar Cells: Recent Advances and Perspectives. Adv. Sci..

[27] Meng X., Ho C.H.Y., Xiao S., Bai Y., Zhang T., Hu C., Lin H., Yang Y., So S.K., Yang S. (2018). Molecular design enabled reduction of interface trap density affords highly efficient and stable perovskite solar cells with over 83% fill factor. Nano Energy.

[28] Sophia J., Muralidharan G. (2014). Preparation of vinyl polymer stabilized silver nanospheres for electro-analytical determination of H_2_O_2_. Sens. Actuat. B-Chem..

[29] Abudabbus M.M., Jevremovic I., Nesovic K., Peric-Grujic A., Rhee K.Y., Miskovic-Stankovic V. (2018). In situ electrochemical synthesis of silver-doped poly(vinyl alcohol)/graphene composite hydrogels and their physico-chemical and thermal properties. Compos. Part B-Eng..

[30] Laghrib F., Ajermoun N., Bakasse M., Lahrich S., El Mhammedi M.A. (2019). Synthesis of silver nanoparticles assisted by chitosan and its application to catalyze the reduction of 4-nitroaniline. Int. J. Biol. Macromol..

[31] Ponnaiah S.K., Periakaruppan P., Vellaichamy B. (2018). New Electrochemical Sensor Based on a Silver-Doped Iron Oxide Nanocomposite Coupled with Polyaniline and Its Sensing Application for Picomolar-Level Detection of Uric Acid in Human Blood and Urine Samples. J. Phys. Chem. B.

[32] Yan X.D., Liu W., Zhou Y., Yuan D., Hu X.W., Zhao W., Zhou G.F. (2019). Improvement of Electro-Optical Properties of PSLC Devices by Silver Nanowire Doping. Appl. Sci..

[33] Wang R., Xu Y., Sors T., Irudayaraj J., Ren W., Wang R. (2018). Impedimetric detection of bacteria by using a microfluidic chip and silver nanoparticle based signal enhancement. Mikrochim. Acta.

[34] Li J.M., Li Y.X., Shahzad S.A., Chen J., Chen Y., Wang Y., Yang M.D., Yu C. (2015). Fluorescence turn-on detection of glucose via the Ag nanoparticle mediated release of a perylene probe. Chem. Commun..

[35] Chen S., Huang D.L., Zeng G.M., Gong X.M., Xue W.J., Li J., Yang Y.Y., Zhou C.Y., Li Z.H., Yan X.L. (2019). Modifying delafossite silver ferrite with polyaniline: Visible-light-response Z-scheme heterojunction with charge transfer driven by internal electric field. Chem. Eng. J..

[36] Calderon-Jimenez B., Johnson M.E., Montoro Bustos A.R., Murphy K.E., Winchester M.R., Vega Baudrit J.R. (2017). Silver Nanoparticles: Technological Advances, Societal Impacts, and Metrological Challenges. Front. Chem..

[37] Zhang X.F., Liu Z.G., Shen W., Gurunathan S. (2016). Silver Nanoparticles: Synthesis, Characterization, Properties, Applications, and Therapeutic Approaches. Int. J. Mol. Sci..

[38] Fairley N. (2020). CASAXPS, Version 2.3.23.

[39] Tougaard S. (1997). Universality classes of inelastic electron scattering cross-sections. Surf. Interface Anal..

[40] Slistan-Grijalva A., Herrera-Urbina R., Rivas-Silva J.F., Avalos-Borja M., Castillon-Barraza F.F., Posada-Amarillas A. (2005). Classical theoretical characterization of the surface plasmon absorption band for silver spherical nanoparticles suspended in water and ethylene glycol. Phys. E.

[41] Chapman R., Mulvaney P. (2001). Electro-optical shifts in silver nanoparticle films. Chem. Phys. Lett..

[42] Ponelyte S., Palevicius A., Guobiene A., Puiso J., Prosycevas I. (2010). Investigation of optical properties of Ag: PMMA nanocomposite structures. Proc. of SPIE.

[43] Cheon J.Y., Kim S.J., Park W.H. (2019). Facile Interpretation of Catalytic Reaction between Organic Dye Pollutants and Silver Nanoparticles with Different Shapes. J. Nanomater..

[44] Parnklang T., Lertvachirapaiboon C., Pienpinijtham P., Wongravee K., Thammacharoen C., Ekgasit S. (2013). H_2_O_2_-triggered shape transformation of silver nanospheres to nanoprisms with controllable longitudinal LSPR wavelengths. RSC Adv..

[45] Persson B.N.J., Liebsch A. (1982). Optical-Properties of Inhomogeneous-Media. Solid State Commun..

[46] Kreibig U., Genzel L. (1985). Optical absorption of small metallic particles. Surf. Sci..

[47] Quinten M., Kreibig U. (1986). Optical properties of aggregates of small metal particles. Surf. Sci..

[48] Khan A.U., Guo Y., Chen X., Liu G. (2019). Spectral-Selective Plasmonic Polymer Nanocomposites across the Visible and Near-Infrared. ACS Nano.

[49] Liu Y., Jordan R.G., Qiu S.L. (1994). Electronic structures of ordered Ag-Mg alloys. Phys. Rev. B.

[50] Tougaard S. (2018). Improved XPS analysis by visual inspection of the survey spectrum. Surf. Interface Anal..

[51] Tougaard S. (2003). Quantitative x-ray photoelectron spectroscopy: Simple algorithm to determine the amount of atoms in the outermost few nanometers. J. Vac. Sci. Technol. A.

[52] Tougaard S. (2005). Algorithm for automatic x-ray photoelectron spectroscopy data processing and x-ray photoelectron spectroscopy imaging. J. Vac. Sci. Technol. A.

[53] Moulder J.F., Stickle W.F., Sobol P.E., Bomben K.D. (1992). Handbook of X-ray Photoelectron Spectroscopy. A Reference Book of Standard Spectra for Identification and Interpretation of XPS Data.

[54] Leiro J., Minni E., Suoninen E. (1983). Study of Plasmon Structure in XPS Spectra of Silver and Gold. J. Phys. F Met. Phys..

[55] Eckardt H., Fritsche L. (1985). Theoretical explanation of the XPS satellite structure of elementary metals: Application to Ag. Solid State Commun..

[56] Pauly N., Yubero F., Tougaard S. (2016). Quantitative analysis of satellite structures in XPS spectra of gold and silver. Appl. Surf. Sci..

[57] Al-Hada M., Gregoratti L., Amati M., Neeb M. (2020). Pristine and oxidised Ag-nanoparticles on free-standing graphene as explored by X-ray photoelectron and Auger spectroscopy. Surf. Sci..

[58] Zhang Z., Jiao J., Jiang Z., Tan D., Fu Q., Bao X., Liu X., Jia J., Xue Q. (2008). Oxygen adsorption on Ag/Si(111)-7 × 7 surfaces. J. Vac. Sci. Technol. A.

[59] Bukhtiyarov V.I., Carley A.F., Dollard L.A., Roberts M.W. (1997). XPS study of oxygen adsorption on supported silver: Effect of particle size. Surf. Sci..

[60] Lopez-Salido I., Lim D.C., Dietsche R., Bertram N., Kim Y.D. (2006). Electronic and geometric properties of Au nanoparticles on Highly Ordered Pyrolytic Graphite (HOPG) studied using X-ray Photoelectron Spectroscopy (XPS) and Scanning Tunneling Microscopy (STM). J. Phys. Chem. B.

[61] Lopez-Salido I., Lim D.C., Kim Y.D. (2005). Ag nanoparticles on highly ordered pyrolytic graphite (HOPG) surfaces studied using STM and XPS. Surf. Sci..

[62] Kim Y.D., Wei T., Wendt S., Goodman D.W. (2003). Ag Adsorption on Various Silica Thin Films. Langmuir.

[63] Shin H.S., Choi H.C., Jung Y., Kim S.B., Song H.J., Shin H.J. (2004). Chemical and size effects of nanocomposites of silver and polyvinyl pyrrolidone determined by X-ray photoemission spectroscopy. Chem. Phys. Lett..

[64] Sinha S., Mukherjee M. (2015). A comparative study about electronic structures at rubrene/Ag and Ag/rubrene interfaces. AIP Adv..

[65] Dolatkhah A., Jani P., Wilson L.D. (2018). Redox-Responsive Polymer Template as an Advanced Multifunctional Catalyst Support for Silver Nanoparticles. Langmuir.

[66] Ferraria A.M., Carapeto A.P., do Rego A.M.B. (2012). X-ray photoelectron spectroscopy: Silver salts revisited. Vacuum.

[67] Egelhoff W.F. (1987). Core-level binding-energy shifts at surfaces and in solids. Surf. Sci. Rep..

[68] Xu L.Q., Wang L., Zhang B., Lim C.H., Chen Y., Neoh K.-G., Kang E.-T., Fu G.D. (2011). Functionalization of reduced graphene oxide nanosheets via stacking interactions with the fluorescent and water-soluble perylene bisimide-containing polymers. Polymer.

[69] Ren L., Wang M., Lu S., Pan L., Xiong Z., Zhang Z., Peng Q., Li Y., Yu J. (2019). Tailoring Thermal Transport Properties of Graphene Paper by Structural Engineering. Sci. Rep..

[70] Beamson G., Briggs D. (1992). High Resolution XPS of Organic Polymers: The Scienta ESCA300 Database.

[71] Scholz M., Schmidt R., Krause S., Scholl A., Reinert F., Wurthner F. (2009). Electronic structure of epitaxial thin films of bay-substituted perylene bisimide dyes. Appl. Phys. A.

[72] Zahn D.R.T., Gavrila G.N., Salvan G. (2007). Electronic and Vibrational Spectroscopies Applied to Organic/Inorganic Interfaces. Chem. Rev..

[73] Scholl A., Zou Y., Jung M., Schmidt T., Fink R., Umbach E. (2004). Line shapes and satellites in high-resolution x-ray photoelectron spectra of large pi-conjugated organic molecules. J. Chem. Phys. Lett..

[74] Emmanouil K., Gawrys P., Zagorska M., Kennou S. (2013). Electronic properties of a perylene bisimide interfaced with gold or aluminum: The influence of the substrate. Microelectron. Eng..

[75] Erbahar D., Susi T., Rocquefelte X., Bittencourt C., Scardamaglia M., Blaha P., Guttmann P., Rotas G., Tagmatarchis N., Zhu X. (2016). Spectromicroscopy of C60 and azafullerene C59N: Identifying surface adsorbed water. Sci. Rep..

[76] Yamamoto S., Bluhm H., Andersson K., Ketteler G., Ogasawara H., Salmeron M., Nilsson A. (2008). In situx-ray photoelectron spectroscopy studies of water on metals and oxides at ambient conditions. J. Phys. Condens. Matter.

[77] Salmeron M. (2018). From Surfaces to Interfaces: Ambient Pressure XPS and Beyond. Top. Catal..

[78] Onoe J., Takeuchi K., Ohno K., Kawazoe Y. (1998). X-ray photoelectron spectroscopy of air-exposed C60 films: Origin of the O 1s core peak. J. Vac. Sci. Technol. A.

[79] Borges B.G.A.L., Veiga A.G., Gioti M., Laskarakis A., Tzounis L., Logothetidis S., Rocco M.L.M. (2018). Surface, interface and electronic properties of F8:F8BT polymeric thin films used for organic light-emitting diode applications. Polym. Int..

